# Longitudinal evaluation of breast tissue in healthy infants: Prevalence and relation to reproductive hormones and growth factors

**DOI:** 10.3389/fendo.2022.1048660

**Published:** 2022-12-01

**Authors:** Marie Lindhardt Ljubicic, Andre Madsen, Emmie N. Upners, Margit Bistrup Fischer, Alexander Siegfried Busch, Hanne Frederiksen, Trine Holm Johannsen, Anders Juul, Casper P. Hagen

**Affiliations:** ^1^ Dept. of Growth and Reproduction, Copenhagen University Hospital - Rigshospitalet, Copenhagen, Denmark; ^2^ International Center for Research and Research Training in Endocrine Disruption of Male Reproduction and Child Health (EDMaRC), Copenhagen University Hospital -Rigshospitalet, Copenhagen, Denmark; ^3^ Department of Medical Biochemistry and Pharmacology, Haukeland University Hospital, Bergen, Norway; ^4^ Department of General Pediatrics, University of Münster, Münster, Germany; ^5^ Department of Clinical Medicine, University of Copenhagen, Copenhagen, Denmark

**Keywords:** thelarche, reproductive hormone, minipuberty, infancy, breast tissue, breast development, PCA, principal component analysis

## Abstract

**Introduction:**

Breast tissue in infancy is a rather undescribed phenomenon. We aimed to describe the prevalence and progression of palpable breast tissue in healthy boys and girls aged 0-1 years and to evaluate clinical markers, individual serum hormone concentrations as well as combined hormone profiles as determinants of the persistence of breast tissue.

**Methods:**

In total, 233 term infants (119 boys, 114 girls) were included and followed from birth until 1 year of age in The COPENHAGEN Minipuberty Study (ClinicalTrials.gov #NTC02784184). Infants were followed up to six times with a clinical examination and serum sampling. Principal component analyses (PCAs) produced combined hormone profiles.

**Results:**

A total of 98% of all infants aged 0-1 year exhibited breast tissue at some point. 50% still had breast tissue present at 0.5-0.6 years in girls and 0.3-0.4 years in boys (‘persistent’). At one year, more girls than boys had breast tissue present (p=0.010). Most clinical and hormonal markers did not differ in infants with/without persistent breast tissue. However, in those with persistent breast tissue, estradiol (first visit, girls, p=0.034), androstenedione, corticosterone, cortisol (first visit, boys, all p<0.050), length (first visit, boys, p=0.030), and testicular volume (0.3-0.4 years, p=0.040) were higher, while IGF-I (0.3-0.4, boys, p=0.033) was lower. In boys, a combined, PCA-derived hormone profile (first visit) was able to predict the persistence of breast tissue (area under the curve=83%) better than any single marker.

**Discussion:**

Palpable breast tissue in infancy is common in both sexes although it persists in significantly more girls than boys at one year of age. Data supports both the early origin of breast tissue (*in utero*- and early postnatal) as well as a role of endogenous hormone production in later development and maintenance.

## Introduction

The appearance of palpable breast tissue is a common occurrence in both girls and boys. In pubertal girls, the attainment of palpable glandular breast tissue marks the onset of puberty, also known as thelarche. In pubertal boys, palpable glandular breast tissue is termed gynecomastia and is observed in approximately 50% of boys (1), with reports ranging from 20-70% (2–4). In infancy, the appearance of breast tissue has also been reported as a common finding with a prevalence of up to 90% in cross-sectional studies (3, 5, 6). However, literature on breast tissue in infancy is scarce. Breast tissue before the first year of life is usually considered harmless (7), but distinguishing between a normal and a pathological occurrence, i.e. precocious puberty, can in some cases be difficult (8, 9) and cases of infants referred to pediatricians due to breast development in infancy are not uncommon (10, 11). To enable the distinction between physiological and pathological breast development, further studies describing not only the prevalence of breast tissue *via* cross-sectional cohorts, but also the development and progression in a healthy cohort of infants followed longitudinally are needed.

Breast tissue and the underlying physiological causes in both pubertal girls and boys have been the focus of several studies. The causes of thelarche in girls, a fundamental pubertal milestone, have been suggested to include ovarian estradiol, but also the peripheral conversion of adrenal androgens to estrogens as well as body composition can cause growth of glandular breast tissue (12–16). Likewise, the cause of gynecomastia in pubertal boys has been suggested to be associated with estrogens, an altered estradiol/testosterone ratio, local sex steroid imbalances, luteinizing hormone (LH), growth hormone as well as insulin-like growth factor-I (IGF-I) (1, 17–21). Endocrine disrupting chemicals have also been suggested to play a role in both sexes, although this topic remains debated (22–25).

Studies of breast tissue in puberty have alluded to the underlying hormone profiles, yet similar studies concerning infancy are rare. Sex differences in the presence of breast tissue later in infancy (26) and breast tissue size have been reported (5, 27, 28) as well as correlations with serum and urinary estradiol in girls (5, 28) and umbilical cord testosterone in boys (27). As such, it still remains unknown whether breast tissue in infancy derives from and is maintained by placental hormone production *in utero*, endogenous hormone production in the infant, or both (26).

Principal component analysis (PCA) is a method of data simplification in which multiple variables are condensed into new variables, e.g. multiple hormones can be condensed into a single hormone profile. Such PCA-derived, combined hormone profiles have, for example, been shown to be better at detecting the presence of breast tissue in pubertal girls better than any single hormone (29).

The current study therefore aimed 1) to describe the prevalence and progression of palpable breast tissue in healthy boys and girls aged 0-1 years of age, and 2) to evaluate individual postnatal clinical markers and hormone concentrations as well as PCA-derived hormone profiles as determinants of the persistence of breast tissue in infancy.

## Materials and methods

### The COPENHAGEN minipuberty study

Healthy expectant mothers and their offspring were recruited as part of The COPENHAGEN Minipuberty Study hosted by the Department of Growth and Reproduction, Rigshospitalet, Copenhagen. A total of 233 full-term infants (119 boys and 114 girls), born term at gestational ages 38+0 to 41+5, were included and followed from birth until 1 year of age. Infants were followed a maximum of six times including a clinical examination and serum sampling. A total of 186 infants completed the entire follow-up period. Further details on the design of the study have previously been described (30). All included infants were healthy and, importantly, not suspected of any endocrinological disorders.

In this current study, all 233 infants had information available on breast tissue at least at one visit. The infants were examined a total of 1,201 times, of which 1193 examinations included information on breast tissue (631 examinations of boys and 562 examinations of girls). These examinations took place between ages 4 days and 16 months of age. Details on clinical markers including body length, body weight, body mass index (BMI), feeding status, testicular volume (in boys), and breast tissue were included as well as serum hormone concentrations. No hormone stimulation tests were performed or medically indicated. Data from patient files on diagnoses given after the study follow-up was unfortunately not available.

Length was determined using a baby length measuring mat (ADE Germany GmbH & Co, Germany) to the nearest 0.5 cm, while weight was determined (without clothes or diapers) on an electronical scale (Baby-scale, Solotop Oy, Finland) to the nearest 0.005 kg. Feeding status was determined using questionnaires and at each visit an infant was grouped as either 1) breast fed only; 2) mixed breast milk, formula, and/or solids; or 3) formula only. Testicular volume was determined by ultrasonography (Hitachi Aloka SSD 500, Mechelen, Belgium) in terms of length and width (mm). An ellipsoid shape was assumed: volume = width x height^2^ x π/6. The presence of breast tissue was determined by palpation (differentiated from pseudo-tissue due to adipose tissue). Both uni- and bilateral breast tissue was defined as the presence of breast tissue. Breast tissue size was measured to the nearest millimeter using a caliper. Breast tissue was deemed transient if it was present at one exam, absent at the next and then present again at a later exam.

Blood samples were acquired from a total of 211 infants (113 boys and 98 girls) comprising 641 samples (338 from boys and 303 from girls). Samples were drawn between five days and 14.2 months of age between 8 a.m. and 4 p.m.

### Hormone assays

Due to limitations of the amount of blood that can be drawn from a healthy infant for research purposes (ethically as well as physically, refer to Busch et al. (30) for details), there was not a complete overlap in the hormones analyzed between the two sexes. In girls, LH, follicle-stimulating hormone (FSH), inhibin B, anti-Müllerian hormone (AMH), estrone (E1), estradiol (E2), sex hormone-binding globulin (SHBG), IGF-I, and insulin-like growth factor-binding protein 3 (IGFBP3) were quantified. In boys, LH, FSH, inhibin B, AMH, testosterone, androstenedione, cortisol, corticosterone, cortisol, 11-deoxycortisol, 17-hydroxyprogesterone, E1-S, SHBG, IGF-I, and IGFBP3 were quantified.

The following analytical methods were used: 1) LH and FSH: time-resolved fluoroimmunometric assays (AutoDELFIA, Perkin Elmer, Turku, Finland, Research Resource Identifier (RRID): For LH: AB_2783737, https://antibodyregistry.org/search.php?q=AB_2783737; for FSH: AB_2783738, https://antibodyregistry.org/search.php?q=AB_2783738) with limits of detection (LOD) of 0.05 IU/L and inter-assay coefficients of variation (CVs) ≤3% for both; 2) Inhibin B: double antibody enzyme-immunometric assay (Inhibin B GenII ELISA, Beckman Coulter, Brea, CA, USA, RRID : AB_2827405, https://antibodyregistry.org/search.php?q=AB_2827405) with an LOD of 3 pg/mL and a CV of <11%; 3) AMH: chemiluminescent immunoassay (Access 2 Immunoassay System, Beckman-Coulter, Brea, CA USA, RRID: AB_2892998, https://antibodyregistry.org/search?q=AB_2892998 for AMH)with an LOD of 0.14 pmol/L and a CV of <6%; 4) SHBG: chemiluminescent assay (Access 2 Immunoassay System, Beckman-Coulter, Brea, CA USA, RRID: AB_2893035, https://antibodyregistry.org/search?q=AB_2893035) with an LOD of 0.33 nmol/L and a CV of ≤10%; 5) estrogens and androgens: in-house liquid chromatography-tandem mass spectrometry (LC-MS/MS) (31, 32) with the following LODs and CVs for three times three different control samples per batch: E1 (LOD: 2.9 pmol/L, CV: 5-7%), E2 (4 pmol/L, 5-7%), testosterone (0.012 nmol/L, 2-6%), androstenedione (0.042 nmol/L, 4-8%), DHEAS (19 nmol/L, 4-10%), cortisol (1.9 nmol/L, 3-6%), corticosterone (0.1 nmol/L, 4-12%), cortisone (0.19 nmol/L, 5-7%), progesterone (0.036 nmol/L, 3-4%), 11-deoxycortisol (0.017 nmol/L, 3-13%), 17-hydroxyprogesterone (0.1 nmol/L, 3-7%), estrone 3-sulphate (0.026 nmol/L, 7-8%); and 6) IGF-I and IGFBP3: chemiluminescence assays (IDS-iSYS, Immuno-diagnostic Systems LTD, Bolton, United Kingdom) with LODs of 10 ng/mL and 80 ng/mL, respectively, and CVs of <8% for both.

For all hormones, concentrations below LOD were reported as LOD/2. The Danish Accreditation Fund (DANAK) for medical examination accredited all the above-mentioned analytical methods according to a European and International standard (the DS/EN ISO 15189).

### Statistical methods

Firstly, Pearson’s Chi-Squared and Fischer’s exact tests were used to test for sex differences in the prevalence of breast tissue at the first and the last exams, respectively. Spearman’s rho was used to examine correlations between continuous markers and size of the largest breast tissue (mm). P-values were considered significant at p < 0.05.

Secondly, part of the study aim was to investigate the underlying hormones associated with breast tissue in infancy. When reviewing the longitudinal data, it was apparent age intervals at 0.3 – 0.4 years (boys) and 0.5 – 0.6 years (girls) were associated with the disappearance of the breast tissue in approximately half of infants in each sex, respectively (still present in 30/62 boys and 31/64 girls). We therefore focused our further analyses on this clinically evident dichotomy and consequently defined breast tissue as persistent if still present at these ages in boys and girls, respectively, and as non-persistent if it had disappeared.

To elucidate the biochemistry underlying the persistence of breast tissue in infancy, we therefore evaluated individual hormones and combined endocrine profiles at the first visit (median age 11 days, range 4-35 days; a reflection of the peri-/neonatal period) and at the ages of 0.3-0.4 and 0.5-0.6 years in boys and girls, respectively (a reflection of endogenous hormones postnatally). At these two timepoints, we 1) by use of Mann Whitney U tests, Pearson’s Chi-squared or Fischer’s Exact tests, identified if any single markers (single hormone concentrations, height, weight, and feeding status) were significantly associated with the persistence of an individual’s breast tissue, and 2) by use of PCAs (described in detail below), examined combined endocrine profiles and tested whether these were able to distinguish between children with persistent vs. non-persistent breast tissue.

PCA is a method of data dimension reduction in which all the variables in a dataset are reduced into a smaller number of new combined ‘variables’ called principal components. By weighing the variance contributed by each of the variables, the principal components produced in a PC analysis account for decreasing amounts of the total dataset variance. To enable the PCAs to account for relevant variance concerning the presence of breast tissue (and not just general interindividual variance attributed to growth, minipuberty as a whole etc), receiver operating characteristic (ROC) curves were used to identify the hormones that were best able to distinguish between infants with persistent and without persistent breast tissue. All hormones with ROC-derived areas under the curve >60% were included in the PCAs. For boys, these hormones were AMH, androstenedione, corticosterone, cortisol, FSH, and IGFBP3, while in girls these hormones were AMH, E2, IGF-I, and inhibin B. In short, PCAs were used to further elucidate the biochemistry (‘endocrinological profiles’) of infants with and without persistent breast tissue and to investigate any differences in hormone concentrations between the two groups.

PCA were subsequently performed for each sex separately. Principal components with an Eigenvalue>1 (‘Kaiser rule’, Eigenvalue is an expression of the standard deviation of a dataset) were deemed viable, and corresponding principal component scores were calculated. These scores each represented a new, combined variable for each child. The abilities of the PC scores to predict the presence of persistent breast tissue were assessed using ROC. AUCs were applied to evaluate the performance of the principal component scores to distinguish between infants with persistent breast tissue from those without: ≥90%: excellent, ≥80-90%: good, ≥70-80%: fair, ≥60-70%: poor, and ≤60%: bad (33, 34). For the principal component scores with the best ability to do so for each sex, the endocrine profiles, i.e. the combination of hormones, underlying this principal component were then evaluated by their correlation coefficients in the given principal component. The correlations coefficients are an expression of the relative importance of the included hormones and values > ± 0.4 were deemed strong correlations (35).

In this study, no correction for multiple testing in the Mann-Whitney U tests was carried out. Dimension reduction by PCA inherently overcomes the problem of multiple testing as the different variables are condensed principal components.

### Ethical considerations

The study has been registered with Clinical Trials.gov (#NTC 02784184). Parents consented in writing as well as orally to the participation of their child The COPENHAGEN Minipuberty Study. The study was approved by the regional Ethics Committees (H-15014876) and the Danish Data Protection Agency (RH-2015-210, I-Suite: 04146).

## Results

### Prevalence and progression of breast tissue in infancy

**Figure 1:**
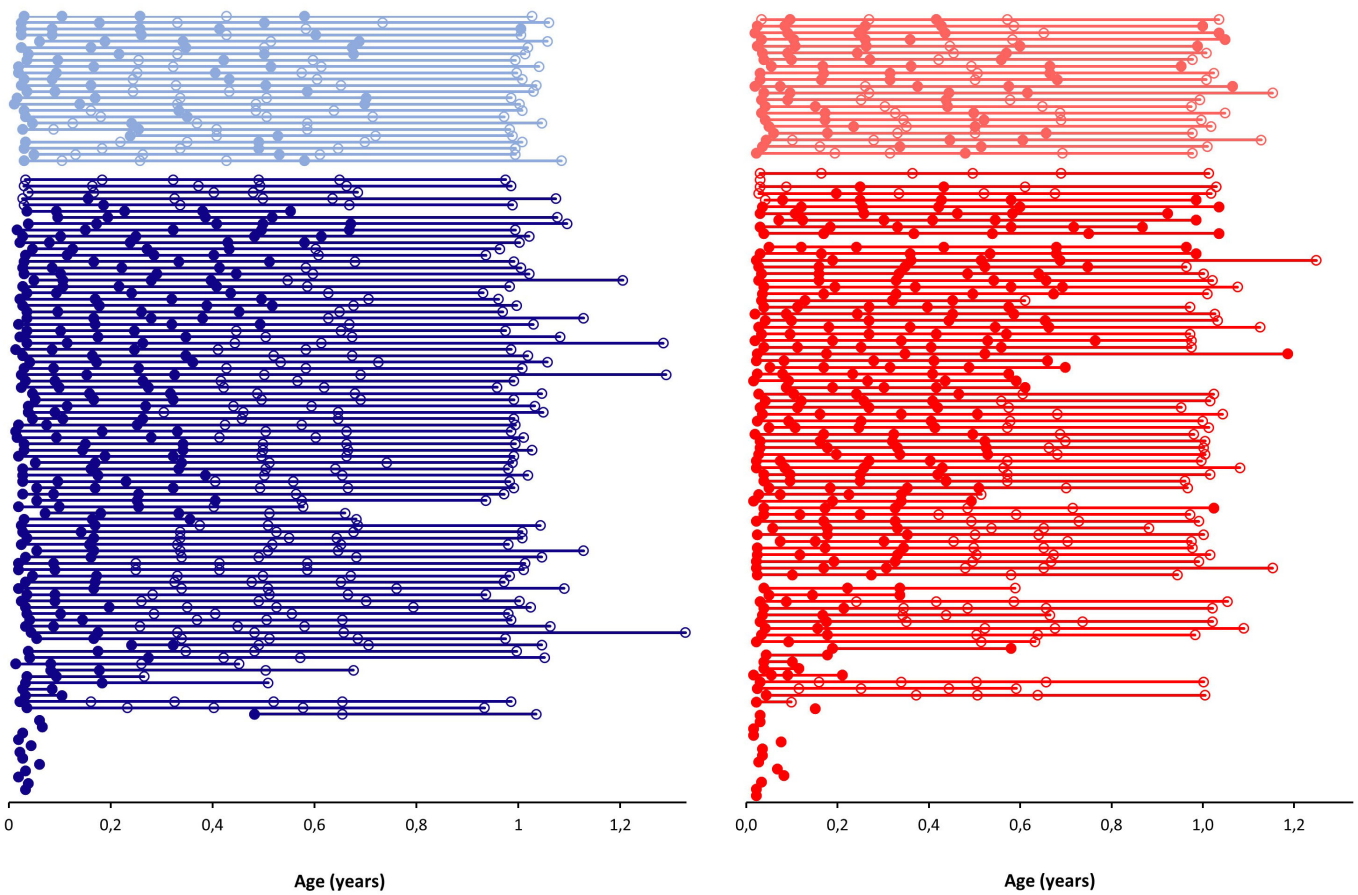
The prevalence of palpable breast tissue during the first year of life in boys (blue) and girls (red) according to age (years). Each connected line represents a single child. Circles indicate examinations; open circles are exams without the presence of palpable breast tissue, while closed circles are exams with the presence of palpable breast tissue. Lighter shades indicate children in which transient breast tissue was present, i.e. the appearance, disappearance and reappearance of breast tissue at consecutive examinations.

The presence of breast tissue was very frequent in both sexes (**Figure 1**). At the initial exam (boys’ median age: 11 days, range: 4-36; girls: 11 days, 5-33) 114 of 116 boys (98%, three had their first examination at a later age) and 108 of 112 girls (96%, two had their first examination at a later stage) exhibited breast tissue. In total, three boys (3%) and two girls (2%) did not have palpable breast tissue at any examination during the first year of life. At the last exam (boys’ age: 12.0 months, 11.2-15.9; girls: 12.0 months, 10.4-15.0), one of 75 boys (1%) and nine of 58 girls (16%) still had palpable breast tissue present, and a marked sex difference was therefore present at the last exam (p = 0.010) but not a the first (p = 0.763).

In 25 of 119 boys (21%) and in 22 of 114 girls (19%) transient breast tissue was observed (**Figure 1**). There was no observed difference between infants with and without transient breast tissue in terms of body weight, length, or BMI (all p > 0.05).

The median diameter of breast tissue at the peak diameter was 13 mm (IQR: 10-16) in boys and 13 mm (9–16) in girls. No apparent associations between concentrations of any of the analyzed hormones and breast tissue size were observed, all Spearman’s rhos were < ± 0.2 (data not shown).

### Determinants of the persistence of breast tissue in infancy

**Figure 2:**
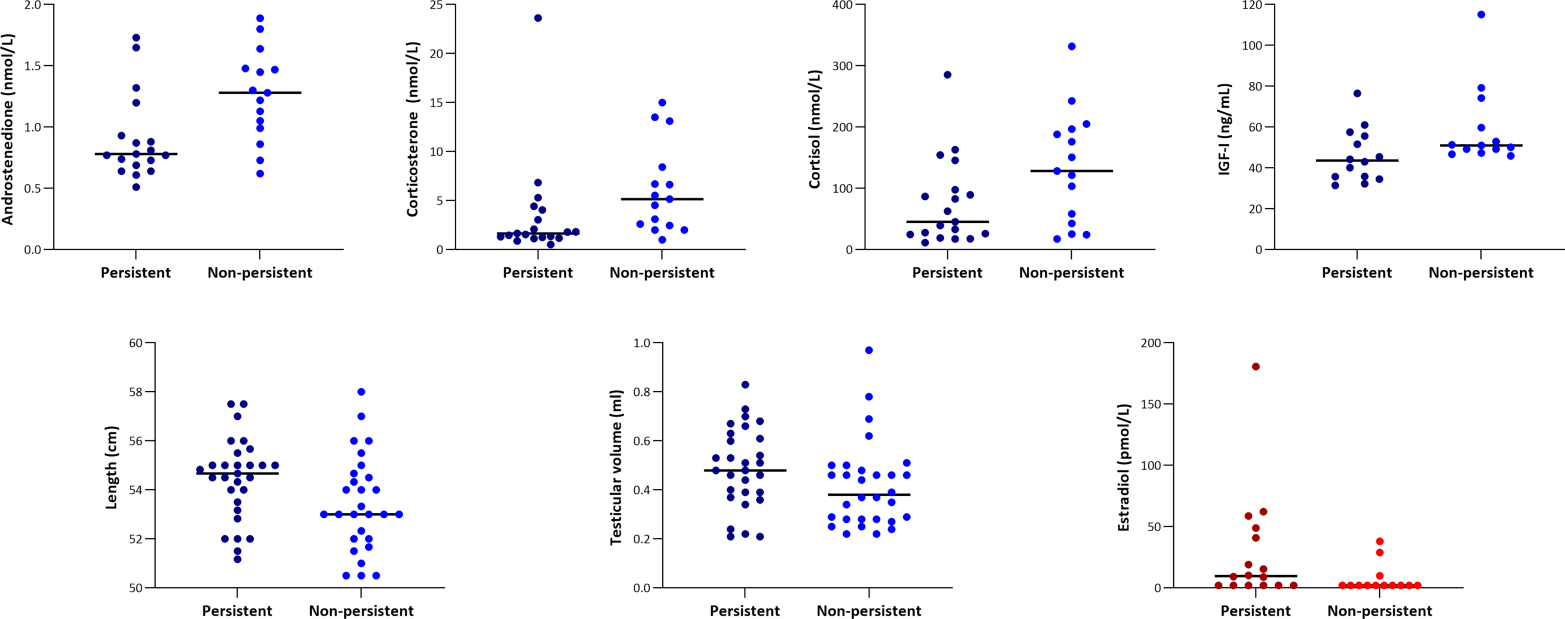
Variables with significant differences between infants with persistent (darker shades) and infants with non-persistent (lighter shades) breast tissue. For the boys (blues), there were significant differences between the groups at the first visit (median age 11 days) for androstenedione, corticosterone, cortisol, and length, while for IGF-I and testicular volume the difference was significant at the exam between 0.3-0.4 years of age. For the girls (reds), estradiol concentrations were significantly different between the groups at the first visit (median age 11 days). Breast tissue was labelled as ‘persistent’ if still present at 0.3-0.4 years of age in boys and at 0.5-0.6 years of age in girls.

The majority of the individual markers measured did not display any significant differences in infants with persistent breast tissue vs. those with non-persistent breast tissue, i.e. palpable breast tissue at these respective ages in each sex (**Tables 1**, **2**). However, in girls, estradiol concentrations measured at the first visit were significantly higher in those with persistent breast tissue (p = 0.034, **Table 2** and **Figure 2**). In boys, serum concentrations of androstenedione, corticosterone, and cortisol measured at the first visit were all significantly higher in boys with non-persistent breast tissue (all p < 0.05, **Table 1** and **Figure 2**). Serum concentrations of IGF-I at 0.3-0.4 years were also significantly higher in those with non-persistent breast tissue (p = 0.033, **Table 1** and **Figure 2**). Conversely, boys with persistent breast tissue were significantly longer at the first examination than those without (p = 0.03, **Table 1** and **Figure 2**). Furthermore, boys with persistent breast tissue had a larger testicular volume at 0.3-0.4 years (p = 0.04, **Figure 2**). Feeding status did not appear to be associated with the persistence of breast tissue (all p > 0.05).

**Table 1:** Median hormone concentrations and anthropometric measures and total counts (n) in boys with persistent and non-persistent breast tissue in infancy at the initial exam (median age 11 days) and at the exam between 0.3-0.4 years of age.

BOYS	11 days				0.3-0.4 years			
	Non-persistent	Persistent	p-value	n	Non-persistent	Persistent	p-value	n
**LH (IU/L)**	4.5	5.2	0.298	31	1.3	1.4	0.782	28
**FSH (IU/L)**	1.6	2.1	0.091	31	0.7	0.7	0.337	29
**Inhibin B (pg/mL)**	206	190	0.535	31	368	287	0.114	22
**AMH (pmol/L)**	552	504	0.246	31	1603	1237	0.116	29
**Androstenedione (nmol/L)**	1.3	0.8	**0.029**	34	0.5	0.3	0.106	29
**Corticosterone (nmol/L)**	5.2	1.7	**0.006**	34	5.8	5.7	0.983	29
**Cortisol (nmol/L)**	129	45.5	**0.046**	34	226	148.	0.600	29
**Cortisone (nmol/L)**	94.0	106	0.903	34	58.5	45.9	0.163	29
**E1-S (nmol/L)**	0.3	0.01	0.213	34	0.01	0.01	0.301	29
**DHEAS (nmol/L)**	672	709	0.521	34	172	112	0.093	29
**Progesterone (nmol/L)**	0.5	0.3	0.238	34	0.1	0.04	0.078	29
**Testosterone (nmol/L)**	4.1	3.8	0.532	34	2.4	1.9	0.060	29
**11-deoxycortisol (nmol/L)**	1.3	1.3	0.849	34	2.3	1.0	0.359	29
**17-hydroxyprogesterone (nmol/L)**	2.3	2.1	0.591	34	1.4	0.7	0.198	29
**SHBG (nmol/L)**	102	89.7	0.561	23	154	169	0.828	25
**IGF-I (ng/mL)**	92	91	0.529	29	51	44	**0.033**	27
**IGFBP3 (ng/mL)**	1758	1951	0.164	29	2155	2095	0.099	27
**Weight (g)**	3607	3960	0.051	57	7246	7073	0.907	58
**Length (cm)**	53.0	54.7	**0.030**	57	66.0	66.2	0.462	58
**Testicular volume (mL)**	0.34	0.34	0.743	57	0.38	0.48	0.030	60

P-values express any differences observed between the two groups. Significant p-values (< 0.05) are highlighted in bold. Decimals have been rounded to nearest clinically meaningful number.

n, total count; LH, luteinizing hormone; FSH, follicle-stimulating hormone; AMH, anti-Müllerian hormone; E1-S, estrone sulphate; DHEAS, dehydroepiandrosterone-sulphate; SHBG, sex hormone-binding globulin; IGF-I, insulin-like growth factor-I; and IGFBP3, insulin-like growth factor-binding protein 3.

**Table 2:** Median hormone concentrations and anthropometric measures and total counts (n) in girls with persistent and non-persistent breast tissue in infancy at the initial exam (median age 11 days) and at the exam between 0.5-0.6 years of age.

GIRLS	11 days				0.5-0.6 years			
	Persistent	Non-persistent	p-value	n	Persistent	Non-persistent	p-value	n
**LH (IU/L)**	0.4	0.7	0.617	34	0.1	0.1	0.806	29
**FSH (IU/L)**	4.3	5.3	0.692	34	4.8	3.8	0.337	29
**Inhibin B (pg/mL)**	37	11	0.183	29	37	50	0.843	22
**AMH (pmol/L)**	2.9	4.6	0.212	30	10.5	29.3	0.565	28
**Estrone (pmol/L)**	14.4	25.1	0.539	29	4.9	4.6	0.356	28
**Estradiol (pmol/L)**	8.4	3.7	**0.034**	29	14.1	21.3	0.299	28
**SHBG (nmol/L)**	87.0	84.5	0.918	30	148.3	144.0	0.730	28
**IGF-I (ng/mL)**	88	88	0.069	28	46	36	0.734	27
**IGFBP3 (ng/mL)**	2206	2023	0.908	28	2238	1847	0.961	27
**Weight (g)**	3553	3330	0.583	62	7507	7768	0.248	64
**Length (cm)**	52.5	52.5	0.277	62	68.0	67.0	0.226	64

P-values express any differences observed between the two groups. Significant p-values (< 0.05) are highlighted in bold. Decimals have been rounded to nearest clinically meaningful number.

n, total count; LH, luteinizing hormone; FSH, follicle-stimulating hormone; AMH, anti-Müllerian hormone; SHBG, sex hormone-binding globulin; IGF-I, insulin-like growth factor-I; and IGFBP3, insulin-like growth factor-binding protein 3.

Combined hormone profiles obtained by PCA are outlined in **Tables 3**, **4**. In boys, the combined hormone profile at the first visit specified by principal component 2 was able to predict the persistence of an infant’s breast tissue (AUC = 83%, i.e. ‘good’). In girls, the best principal component at the first visit displayed fair abilities (AUC = 72%) to predict the persistence of the breast tissue based on the hormones from the first visit. PCA-derived hormone profiles at ages 0.3-0.4 or 0.5-0.6 years in each sex respectively displayed AUCs of 46 – 57%, i.e. ‘bad’. However, the second principal component in boys at this age had a positive predictive value of 100%.

**Table 3:** Principal components and correlation coefficients for hormone concentrations in boys at the initial exam (median age: 11 days) and at the exam between 0.3-0.4 years of age.

BOYS	11 days		0.3-0.4 years	
	PC1	PC2	PC1	PC2
*Correlations coefficients*				
**Corticosterone**	-0.610	0.233	-0.537	0.231
**Androstenedione**	-0.169	0.445	-0.476	0.005
**Cortisol**	-0.643	0.164	-0.533	0.277
**FSH**	-0.122	-0.592	-0.190	-0.686
**IGFBP3**	-0.239	-0.384	0.334	-0.059
**AMH**	0.337	0.472	0.231	0.639
*PCA-derived values*				
**Variance explained (%)**	35	25	39	24
**Cumulative variance (%)**	35	60	39	63
**Eigenvalue**	2.11	1.47	2.33	1.47
*ROC results*				
**AUC (%)**	53	83	46	57
**PPV (%)**	50	73	64	100
**NPV (%)**	72	88	67	61
**ACCURACY (%)**	64	82	65	65

The ability of principal component-derived combined hormone scores to distinguish between boys with persistent and non-persistent breast tissue was evaluated by Receiver operating curves (ROC).

PC, principal component; FSH, follicle-stimulating hormone; IGFBP3, insulin-like growth factor-binding protein 3; AMH, anti-Müllerian hormone; PCA, principal component analysis; ROC, receiver operating characteristics; AUC, area under the curve; PPV, positive predictive value; and NPV, negative predictive value.

**Table 4:** Principal components and correlation coefficients for hormone concentrations in girls at the initial exam (median age: 11 days) and at the exam between 0.5-0.6 years of age.

GIRLS	11 days		0.5-0.6 years	
	PC1	PC2	PC1	PC2
*Correlations coefficients*				
**Estradiol**	0.517	-0.222	0.516	0.102
**IGF-I**	0.170	0.962	-0.175	0.949
**Inhibin B**	0.588	0.067	0.697	-0.035
**AMH**	0.598	-0.147	0.467	0.296
*PCA-derived values*				
**Variance explained (%)**	56	25	46	25
**Cumulative variance (%)**	56	81	46	71
**Eigenvalue**	2.22	0.99	1.85	1.02
*ROC results*				
**AUC (%)**	72	63	51	57
**PPV (%)**	75	71	63	67
**NPV (%)**	77	61	67	60
**ACCURACY (%)**	76	64	64	64

The ability of principal component analysis (PCA)-derived combined hormone scores to distinguish between girls with persistent and non-persistent breast tissue was evaluated by Receiver operating curves (ROC).

PC, principal component; IGF-1I insulin-like growth factor-I; AMH, anti-Müllerian hormone; PCA, principal component analysis; ROC, receiver operating characteristics; AUC, area under the curve; PPV, positive predictive value; and NPV, negative predictive value.

**Figure 3:**
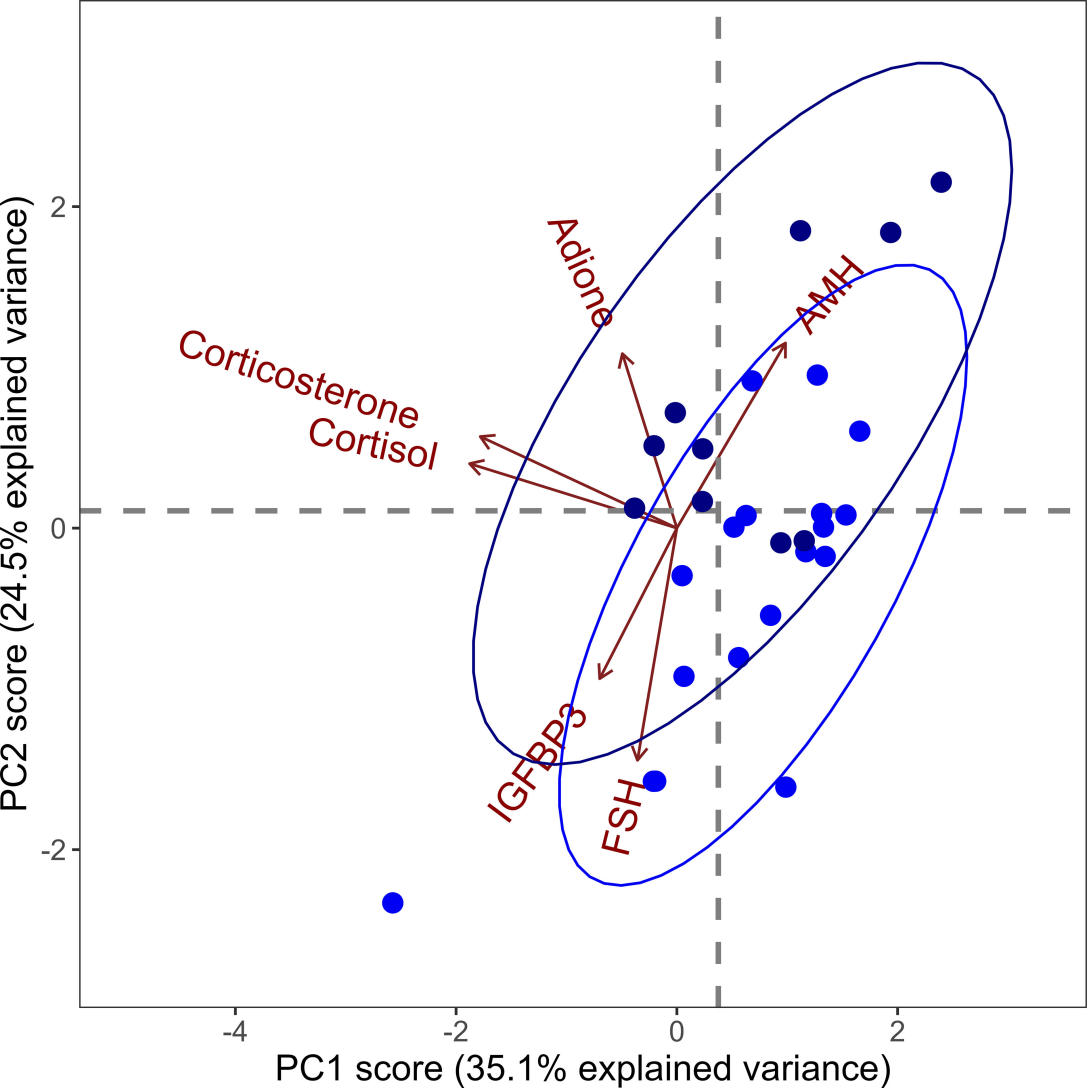
Principal component (PC) biplot for PC1 and PC2 based on hormone concentrations of anti-Müllerian hormone (AMH), adione (androstenedione), corticosterone, cortisol, insulin-like growth factor-binding protein-3 (IGFBP3) and follicle-stimulating hormone (FSH) in boys at the first visit (median age 11 days). These two principal components account for a total of 59.6% of the dataset variance. Arrows (i.e. vectors) represent the correlation coefficients of the hormones with the principal components, and these should be interpreted horizontally for PC1 and vertically for PC2. While cortisol and corticosterone were almost aligned in the horizontal plane, indicating a strong correlation with PC1, adione was almost aligned vertically, indicating a strong correlation with PC2. Darker blue dots represent boys with persistent breast tissue, while lighter blue dots represent boys with non-persistent.

In boys, the correlation coefficients in the best PCA model (first visit), FSH, AMH, and androstenedione showed strong correlations to the profile (all > ± 0.4). These correlations and the ability of the hormone profile to distinguish between breast tissue persistence are both similarly visible in the corresponding biplot, which visualizes the relative and separate clustering of those with and without persistent breast tissue (**Figure 3**). In girls, there were strong correlations (> ± 0.4) for AMH, inhibin B and estradiol displayed in predominant (best) principal component, PC1 (first visit).

## Discussion

In this cohort study of 233 healthy infant boys and girls followed longitudinally throughout the first year of life, palpable breast tissue was a very common finding. Shortly after birth there was no difference in the prevalence between the sexes, while at one year of age, breast tissue was still present in 16% of girls but only in 1% of boys. Differences in adrenal androgens, estradiol, body length, and principal component analysis-derived combined hormone profiles shortly after birth alluded to the breast tissue originating either *in-utero* or in early postnatal life, while differences in IGF-I and testicular volume later in infancy indicated that endogenous hormone production also plays a role in the maintenance of breast tissue in infancy.

In total, 98% of all infants had palpable breast tissue at some point in time during the first year of life, which is in line with or slightly higher than previous reports (3, 6, 36). At one year of age, there was a marked sex difference with the presence of breast tissue being more frequent in girls. However, transient breast tissue was a phenomenon present in a substantial proportion of both boys and girls. This has been scarcely described in infancy (37), but is a well-known occurrence in pubertal boys (gynecomastia) and girls (transient thelarche) (4, 13, 16, 38). These observations are important to note as they may aid clinicians in distinguishing between the normal physiological occurrence of breast tissue in infancy and premature thelarche (8, 9, 11, 39). Thus, in both boys and girls with breast tissue (transient or not) present during the first year of life as the sole sign of puberty, precocious puberty seems highly unlikely. Aside from the clinical application of our findings, breast tissue has also been suggested as a non-invasive means of monitoring exogenous estrogenization effects from environmental chemicals (40). Yet given the high prevalence of palpable breast tissue, as well as the transient nature in both sexes, breast tissue in early infancy as a proxy of chemical exposure would have inherent limitations. It is important to note that the high prevalence of breast tissue in both sexes in this study may reflect different aspects, including but not limited to: the method of palpation vs. ultrasound; demographic-specific results that may arise due to e.g. differences in feeding patterns (high degree of breast-fed v bottle-fed (41) and/or differences in exposure to endocrine disrupting chemicals (42, 43) both across and within countries. Moreover, it is important to note that while the current study elucidates that the presence of breast tissue, in both sexes, is a normal, physiological phenomenon in infants younger than 1 year of age, it does not investigate whether persistent breast tissue in infancy is a risk marker of future precocious puberty.

Altogether, the analyses concerning the underlying etiology of breast tissue development in infancy alluded to both *in utero*/early postnatal factors and later endogenous hormone production in the infant. Specifically, adrenal androgens and length in boys and estradiol concentrations in girls in early postnatal life indicated that *in utero* milieu and/or very early postnatal hormone production play a role in the initial development of breast tissue. Moreover, the ability of the PCA-derived, combined hormone profiles shortly after birth (first visit) to distinguish between infants with and without persistent breast tissue was further indication of the pre- and/or early post-natal origin. The correlation coefficients in the PCA analyses confirmed the role of androstenedione as well as FSH and AMH in regulating infant breast tissue boys. In girls, AMH, inhibin B, and estradiol were also strongly correlated at this age. Altogether, the data supports previous studies reporting that most infants were born with palpable breast tissue (26, 27, 44), which makes the fetal origin, at least in part, likely. In line with this, maternal estrogens have been mentioned in multiple studies as reasons for breast tissue development in infancy (40, 45). However, our study design, which lacks a perinatal examination, does not allow for an absolute distinction between the pre- and postnatal periods.

While our study may allude to a pre-/early postnatal origin of breast tissue in infants, the mere fact that some infants had breast tissue present several months after birth while others did not, as well as the transient phenomenon in some infants, indicates that endogenous hormone production plays a role in the maintenance of breast tissue as well (26). This has also been observed in other studies in which estradiol has been found to be associated with breast tissue diameter and development (5, 28). Estradiol production in girls has also been suggested as the reason for the marked sex difference in the latter part of infancy (28), a sex difference which was also observed in our study. This is likely ovarian estradiol with the known activation of the hypothalamic-pituitary-gonadal axis during minipuberty, although adrenal origin as well as peripheral aromatase action in the fatty tissue also cannot be ruled out.

The ability of testicular volume at 0.3-0.4 years of age to predict breast tissue persistence in our data also heavily suggests that the testicular hormone production is, at least in part, responsible for the maintenance of breast tissue in infant boys. Moreover, IGF-I at age 0.3-0.4 years was also found to be significantly lower in infant boys with persistent breast tissue. However, this is in contrast to reports of higher concentrations of IGF-I in boys with pubertal gynecomastia (1). While the overall performances of the combined hormone profiles produced by PCA later in infancy were not statistically meaningful, the PCA model summarizing newborn endocrine profiles in boys exhibited a positive predictive value of 100%, implying that endocrine profile scoring in this way with great certainty can predict the persistence of infant breast tissue. Although the ROC performances overall were poor, there were still indications that the combined hormone profiles later in infancy play a role in the persistence of breast tissue.

We applied PCA methodology to elucidate the discriminatory capabilities of combined hormone profiles on the persistence of infant breast tissue, and found that combined hormone profiles, primarily at the first visit but also later in infancy, were closely associated with persistence. Notably, in this study, PCA was used to investigate normal physiology rather than for a proposed direct clinical application. The PCA method is routinely used to assess dichotomic clustering in dataset variance; however, its application in endocrinological research is rather novel. The major advantage is its ability to condense multiple variables into a single principal component (‘variable’) that can allude to the underlying biochemistry of the phenotype in question. Summarizing the contributions of all included feature variables as one or two principal components eliminates the concerns of multiple testing and p-value overestimation. Although the hormone profiles investigated by PCA in this current study may not provide immediate clinical utility, characterizing pediatric development trajectories is an important aspect of pediatric endocrinology.

Aside from the novel use of PCA, the strengths of this study included: 1) the design of the cohort of healthy infants followed longitudinally allowing for the description of both prevalence and the progression of breast tissue in infancy; 2) the rather large study size of 233 infants; 3) the frequent serum sampling; and 4) the use of highly sensitive hormone analytical methods. However, the study also had limitations which included: 1) due to limitations in serum sample volumes, estrogens were not quantified in boys and androgens were not quantified in girls; 2) all infants were Caucasian which restricts generalizability; 3) the measurement of the size of the breast tissue can be difficult and interobserver variation has previously been noted in both the current cohort (30) as well as other cohorts (5). This interobserver variation, which may also have been affected by infant weight/body size, could possibly have been limited by the use of breast ultrasound, which reportedly has a small intra- and interobserver variation (46); 4) infants contributed with multiple observations in the Spearman’s rho analyses, yet the distribution of the multiple samples was random and there was no reason to believe that those who included more observations were outliers/at ends of the given scales; 5) the cohort was recruited in affluent areas of Copenhagen and consequently very few infants were formula-fed vs. breast milk-fed, which may have hidden true differences between the groups; and 6) *post-hoc* correction for multiple testing was not performed as it would have obscured the significant associations presented in the study. The study was exploratory in nature, and, as such, the weight attributed to a single significant value was limited. Additionally, only associations with a biological foundation and/or previously described by other groups were examined.

In conclusion, palpable breast tissue in infancy is very common in both sexes although it persists in significantly more girls than boys at one year of age. As in puberty, transient breast tissue (i.e. the appearance, disappearance, and reappearance) was very common in both sexes. The data presented on a whole supports both the early origin of breast tissue (*in utero*- and early postnatal) as well as a role of endogenous hormone production in later development and maintenance. Altogether, the presence of palpable breast tissue throughout the first year of life is a normal phenomenon in both sexes and may not alone warrant further endocrinological workup, although individual evaluation and management is important.

## Data availability statement

The datasets presented in this article are not readily available because Danish/EU data protection legislation as well as ethical considerations do not allow for it. Requests to access the datasets should be directed to MLL, marie.lindhardt.ljubicic@regionh.dk.

## Ethics statement

The studies involving human participants were reviewed and approved by The Regional Ethics Committees (H-15014876). Written informed consent to participate in this study was provided by the participants’ legal guardian/next of kin.

## Author contributions

ML, EU, MF, and AB all took part in study design, data collection, data processing, interpretation of data, and writing of the manuscript. HF, TJ, AJ, and CH took part in study design, data processing, interpretation of data, and writing of the manuscript. AM took part in data processing, interpretation of data, and writing of the manuscript. Lastly, ML and AM carried out all statistical analyses. All authors contributed to the article and approved the submitted version.

## Funding

The COPENHAGEN Minipuberty Study received funding from: 1) The Absalon Foundation, no F-23653-01 (ML); 2) Aase og Ejnar Danielsens Fond: no. 10-001874 (AJ); 3) Candy Foundation, no 2017-224 og 2020-344 (EU); 4) EDMaRC: no. 1500321/1604357 (AB); 5) The Research Council of Capital Region of Denmark no. E-22717-11 (AJ); and 6) The Research Council of Rigshospitalet (AU, AB, ML) (nos. E-22717-12, E-22717-07, E-22717-08). Additionally, AB is funded by the Deutsche Forschungsgemeinschaft (DFG, German Research Foundation) – 464240267.

## Acknowledgments

The authors thank all participants and their parents for their contribution to this study.

## Conflict of interest

The authors declare that the research was conducted in the absence of any commercial or financial relationships that could be construed as a potential conflict of interest.

## Publisher’s note

All claims expressed in this article are solely those of the authors and do not necessarily represent those of their affiliated organizations, or those of the publisher, the editors and the reviewers. Any product that may be evaluated in this article, or claim that may be made by its manufacturer, is not guaranteed or endorsed by the publisher.
